# Screening and Evaluation of Active Compounds in Polyphenol Mixtures by a Novel AAPH Offline HPLC Method and Its Application

**DOI:** 10.3390/foods12061258

**Published:** 2023-03-16

**Authors:** Zhaoyang Wu, Guanglei Zuo, Soo-Kyeong Lee, Sung-Mo Kang, Sang-Youn Lee, Saba Noreen, Soon-Sung Lim

**Affiliations:** 1 Department of Food Science and Nutrition, Hallym University, 1 Hallymdeahak-gil, Chuncheon 24252, Republic of Korea; 2 Pharmaceutical Informatics Institute, College of Pharmaceutical Sciences, Zhejiang University, Hangzhou 310058, China; 3 Innovation Center of Translational Pharmacy, Jinhua Institute, Zhejiang University, Jinhua 321016, China; 4 Institute of Korean Nutrition, Hallym University, 1 Hallymdeahak-gil, Chuncheon 24252, Republic of Korea; 5 Institute for Liver and Digestive Diseases, Hallym University, Chuncheon 24252, Republic of Korea

**Keywords:** polyphenols, offline HPLC, AAPH, *Lepechinia meyenii* (Walp.) *Epling*

## Abstract

In this study, we developed a novel offline high-performance liquid chromatography (HPLC) method based on 2,2′-azobis(2-amidinopropane) dihydrochloride (AAPH) radicals for antioxidant screening in 20 polyphenolic compounds and used the Trolox equivalent antioxidant capacity assay to evaluate their antioxidant activity. Compared to the existing offline HPLC methods based on 2,2′-azino-bis(3-ethylbenzothiazoline-6-sulfonic acid (ABTS) and 2,2-diphenyl-1-picrylhydrazyl (DPPH), the offline HPLC method based on the AAPH radical is more sensitive. Additionally, we applied this method to *Lepechinia meyenii* (Walp.) *Epling* extract and screened out seven antioxidants, caffeic acid, hesperidin, rosmarinic acid, diosmin, methyl rosmarinate, diosmetin, and n-butyl rosmarinate, which are known antioxidants. Therefore, this study provides new insights into the screening of antioxidants in natural extracts.

## 1. Introduction

In recent years, the incidence of chronic diseases such as diabetes, obesity, cardiovascular and cerebrovascular diseases, and cancer has increased annually, resulting in a serious disease burden [1]. Growing evidence supports the crucial role that oxidative stress plays in the development of tissue injury, leading to a range of pathologies, all of which are characterized by a change in oxidative status [2]. Oxidative stress refers to a condition where cell membrane components, such as proteins, nucleic acids, and lipids, are damaged by oxidants through non-enzymatic means [3]. Therefore, antioxidants can act as key factors against oxidative stress to reduce the incidence of chronic diseases.

Indeed, multiple preclinical and clinical studies indicate that lipid peroxidation products play a role in several pathological conditions, including inflammation, atherosclerosis, diabetes, aging, neurodegenerative diseases, and cancer [4]. 2,2′-Azobis(2-amidinopropane) dihydrochloride (AAPH) is well known to strongly induce lipid peroxidation in members, resulting in viability reduction and upregulation of reactive oxygen species generation [5,6]. Currently, the most widely used offline screening method for antioxidants is based on 2,2′-azino-bis(3-ethylbenzothiazoline-6-sulfonic acid (ABTS) and 2,2-diphenyl-1-picrylhydrazyl (DPPH) radicals, and at present, there is no offline screening method based on lipid peroxidation-related radicals. The electron transfer mode of DPPH and ABTS radicals is single-electron transfer, while the electron transfer mode of AAPH radicals is hydrogen atom transfer [7]. Based on the different electron transfer patterns, the compounds screened from natural products are also different. Therefore, it is necessary to develop an offline method based on AAPH radicals to screen antioxidants to delay the cell damage caused by lipid peroxidation products.

Phenolic compounds are a relatively important class of compounds among the many secondary metabolites of plants. It refers to a class of compounds formed by replacing the hydrogen atoms on the benzene rings in aromatic hydrocarbons with hydroxyl groups. More than 8000 polyphenols have been identified, which can be further categorized into three main groups [8,9,10]. Flavonoids are a group of polyphenolic secondary metabolites occurring in plants, with C6-C3-C6 as the basic structure, and include quercetin, catechin, taxifolin, and apigenin [11]. Phenolic acids are a group of compounds that consist of a phenolic ring and an organic carboxylic acid function (C6-C1 skeleton), including hydroxybenzoic acids (gallic acid) and hydroxycinnamic acids (caffeic acid). Stilbenes are a group of natural compounds that are characterized by the presence of a central trans-stilbene core structure, which consists of two phenyl rings linked by a double bond, and the typical compound is resveratrol [12].

Natural products are an important source of antioxidants, especially herbal medicines. *Lepechinia meyenii* (Walp.) *Epling* (*L. meyenii*), a member of the *Lamiaceae* family, is indigenous to Argentina, Bolivia, and Peru, and its herbal infusion is commonly used in Peru as a traditional medicine to treat a range of conditions, including diabetes, cough, inflammation, diarrhea, spasms, stomach discomfort, and joint and stomach pain [13]. In our previous study, *L. meyenii* extract was found to have good antioxidant activity, and seven main components were isolated and identified from its extract; however, their anti-lipid peroxidation activity was unknown, which prompted us to use offline HPLC to further clarify the anti-lipid peroxidation activity and composition of *L. meyenii* [14].

In this study, we developed a novel offline high-performance liquid chromatography (HPLC) method based on AAPH radicals for the antioxidant screening of 20 polyphenolic compounds and used the Trolox equivalent antioxidant capacity assay to evaluate their antioxidant activity. The developed method was then applied to *L. meyenii*, and the results obtained were compared with those obtained by the existing DPPH offline HPLC and ABTS offline HPLC methods.

## 2. Materials and Methods

### 2.1. Reagents and Plants

2,2′-Azobis (2-amidinopropane) dihydrochloride (AAPH), 2,2′-azino-bis(3-ethylbenzothiazoline-6-sulfonic acid (ABTS), 2,2-diphenyl-1-picrylhydrazyl (DPPH), apigenin, caffeic acid, (+)-catechin, p-coumaric acid, 3,4-dihydroxy-L-phenylalanin, 2,5-dihydroxybenzoic acid, 3,4-dihydroxybenzoic acid, 2,4-dihydroxycinnamic acid, 3,4-dihydroxyhydrocinnamic acid, formic acid, gallic acid, 4-hydroxyphenylacetic acid, 2-hydroxycinnamic acid, 4,4′-methylenediphenol, potassium chloride, potassium persulfate, potassium phosphate monobasic, quercetin, resveratrol, rosmarinic acid, sinapic acid, syringic acid, sodium hydrogen phosphate, sodium chloride, taxifolin, and Trolox were purchased from Sigma-Aldrich Chemical Co. (St. Louis, MO, USA). Methanol was purchased from J. T. Baker Co. (Phillipsburg, NJ, USA) for sample preparation and analysis. Ultrapure water used in this study was produced using a Milli-Q water purification system (Millipore Co., Bedford, MA, USA).

The aerial parts of *L. meyenii* were gathered from Lima in 2015 and verified by Paul H. Gonzales Arce (P.H.G.A.). The dried samples (L-2015-A30), extract (L-2015-A30E), and separated compounds (L-2015-A30C1-7) were placed at the Center for Efficacy Assessment and Development of Functional Foods and Drugs at Hallym University.

### 2.2. Preparation of Single Standard, Mixed Standards and L. meyenii Extract

First, 20 kinds of 50 mmol methanol solutions of polyphenol standards were prepared and stored at 4 °C in the dark for future experiments. Then, using the same volume, the 20 standard solutions were mixed to prepare a 2.5 mmol mixed standard solution, which was diluted to 320 µmol in methanol and stored at −20 °C in the dark for future use. The *L. meyenii* extract and active compounds isolated from it used in this study were obtained from previous research, prepared as 1 mg/mL methanol, and stored at −20 °C in the dark for future use [14].

### 2.3. Optimization of AAPH Radical Generation Condition

AAPH radicals were generated under heating conditions. In this study, we investigated the effects of water bath temperature (65, 70, 75, 80, 85 °C), heating time (20, 30, 40, 50, 60 min), and concentration of AAPH (500, 750, 1000, 1250, and 1500 µmol in PBS; pH = 7.4) on the production of AAPH radicals using a single factor experiment, and the consumption of Trolox was used as a quantitative standard. While investigating other factors, this study used 75 °C, 30 min, and 500 µmol to control for variables. Trolox content was quantified using a standard curve (50–500 µmol) and detected at 291 nm using a UV spectrophotometer (UV1601, SHIMADZU, Kyoto, Japan) [15].

### 2.4. HPLC Analysis

The mixed standard was analyzed using equipment from Agilent Technologies (Santa Clara, CA, USA). The setup consisted of a G1311A pump, a G1329A automated sample injector, a G1316A column oven kept at 30 °C, and a G1314D detector. The HPLC mobile phases used were acidic water (0.1% formic acid; A) and methanol (B). The mixed standards were analyzed at 254 nm and separated with a flow rate of 0.7 mL/min using an Eclipse XDB-C18 column (250 × 4.6 mm, 5 µm). The separation process was as follows: 10% B from 0–5 min, 10–50% B from 5–40 min, 50–100% B from 40–55 min, and 100% B from 55–60 min. The *L. meyenii* samples were also analyzed at 254 nm and separated with a flow rate of 0.7 mL/min using an Eclipse XDB-C18 column (250 × 4.6 mm, 5 µm), with the separation process being 10–100% B at 0–20 min, and 100% B at 20–26 min [13].

### 2.5. Screening Antioxidants from Mixed Standards and Extract Using AAPH Offline HPLC

Briefly, a mixture of 300 µL of the AAPH solution (16 mmol in PBS with a pH of 7.4) and 300 µL of the mixed standard solution (320 µmol in methanol) or extract (1 mg/mL in methanol) was incubated for 50 min at 65, 75, and 85 °C. Therefore, the reaction solution with an injection volume of 10 µL was analyzed to HPLC. As a blank, the AAPH solution was replaced with PBS when incubating with the mixed standards or extract to form an AAPH free group. Compared to the AAPH free group, compounds with reduced peak areas in the AAPH group were assigned as having potential antioxidant activity. In addition, in order to study the effect of heating temperature on polyphenols, we set up a group, a mixture of 300 µL of PBS and 300 µL of the mixed standard solution or extract without heating, as a control group. Peak area reduction by heating and AAPH radicals was calculated as the following Formulas (1) and (2), respectively:

Peak reduction by heating = (A _control_ − A _AAPH free group_)/A _control_ × 100% (1)

Peak reduction by AAPH radicals = (A _AAPH free group_ − A _AAPH group_)/A _AAPH free group_ × 100% (2)

### 2.6. Screening Antioxidants from Mixed Standards and Extract Using ABTS Offline HPLC

The ABTS radical (ABTS^+^) solution was prepared by mixing 11 mg ABTS, 9.5 mg potassium persulfate, and 300 mL distilled water. ABTS working solution was prepared in the dark by incubation for 16 h at 25 °C. Briefly, 200 µL of ABTS working solution prepared before and 20 µL of the mixed standard solution (320 µmol in methanol) or extract (1 mg/mL in methanol) were mixed and incubated for 6 min at 25 °C in the dark. Therefore, the reaction solution, with an injection volume of 10 µL, was analyzed by HPLC. As a blank, the ABTS solution was replaced with water when incubating with the mixed standards or extract to form an ABTS^+^ free group. Compared to the ABTS^+^ free group, compounds with reduced peak areas in the ABTS^+^ group were assigned as having potential antioxidant activity [16]. Peak area reduction by ABTS radicals was calculated using the following Formula (3):

Peak reduction by ABTS radicals = (A _ABTS free group_ − A _ABTS group_)/ A _ABTS free group_ × 100% (3)

### 2.7. Screening Antioxidants from Mixed Standards and Extract Using DPPH Offline HPLC

Briefly, 150 µL of DPPH solution (2.5 mg/mL in methanol) and 50 µL of the mixed standard solution (320 µmol in methanol) or extract (1 mg/mL in methanol) were mixed and incubated for 30 min at 37 °C. Therefore, the reaction solution, with an injection volume of 10 µL, was analyzed by HPLC. As a control, the DPPH solution was replaced with methanol when incubating with the mixed standards or extract to form a DPPH free group. Compared to the DPPH free group, compounds with reduced peak areas in the DPPH group were assigned as having potential antioxidant activity [16]. Peak area reduction by DPPH radicals was calculated using the following Formula (4):

Peak reduction DPPH radicals = (A _DPPH free group_ − A _DPPH group_)/A _DPPH free group_ × 100% (4)

### 2.8. The Trolox Equivalent Antioxidant Capacity (TEAC) Assay

ABTS^+^ solution was prepared as part 2.6. Ten microliters of sample (0.5 mmol in methanol) was added to a 96-well plate with 290 μL of ABTS^+^ solution and incubated in the dark for 10 min at 25 °C, which was detected by an EL800 microplate reader (Bio-Tek Instruments, Winooski, VT, USA) at the absorbance of 750 nm. The standard results are expressed as µmol Trolox equivalents [17].

### 2.9. Statistical Analysis

The TEAC assay was performed in triplicate, and the results are presented as the means ± standard deviations (SDs). One-way ANOVA with Tukey’s multiple comparison test was used to compare the differences in Trolox consumption using Pearson’s correlation coefficients with SPSS software (Version 25; IBM, New York, NY, USA).

## 3. Results and Discussion

### 3.1. Optimization of AAPH Radical Generation Condition

**Figure 1:**
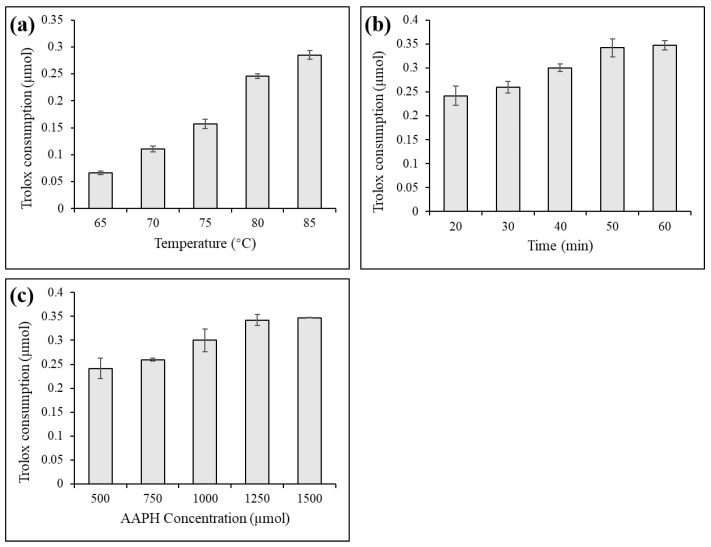
Effects of bath temperature (**a**), heating time (**b**) and AAPH concentration (**c**) on AAPH radical production.

As shown in Figure 1, the water bath temperature, heating time, and AAPH concentration were key factors affecting the generation of AAPH radicals. With the increase in the temperature of the water bath, the prolongation of the heating time, the increase in the concentration of AAPH, and the consumption of Trolox increased, which represented an increase in the generation of AAPH radicals. Therefore, a heating time of 50 min was suitable. When the Trolox concentration was 500 µmol, AAPH at a concentration of 1250 µmol was sufficient. As the concentration of each standard in the mixed polyphenols was 320 µmol, according to the scale conversion, 16 mmol AAPH was used to screen antioxidants from the mixed polyphenols for future experiments.

**Figure 2:**
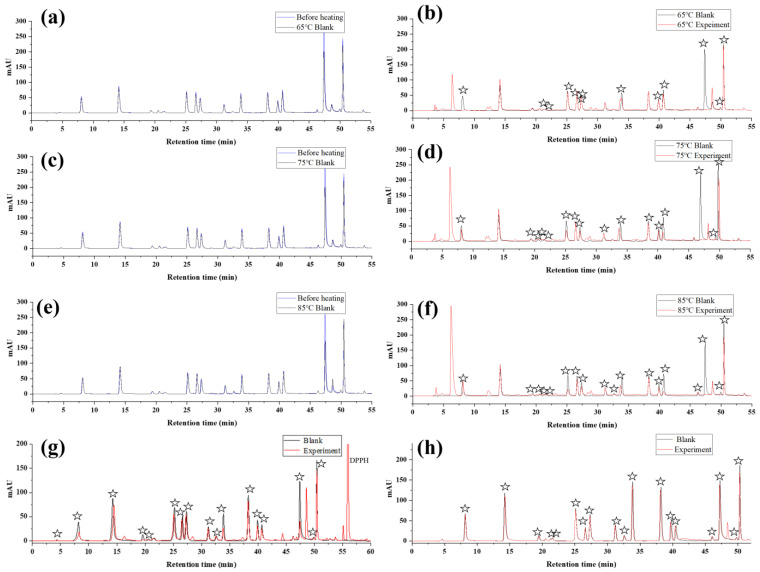
Thermal stability of polyphenols at different temperatures (**a**,**c**,**e**) and results of screening antioxidants based on AAPH radicals (**b**,**d**,**f**), DPPH radicals (**g**) and ABTS radicals (**h**) from mixed polyphenols. The pentagram in figure represents a peak reduction of the compound based on a reaction with radicals.

Phenolic compounds are unstable, and they easily decompose during heating, which was also confirmed by previous research results [18]. Therefore, in this study, we selected 20 phenolic compounds that were common in plant extracts. Then we investigated the thermal stabilities of phenolic compounds at different temperatures (Figure 2a,c,e), and the generation of AAPH radicals (Figure 2b,d,f). The results are shown in Figure 2 and Table 1. In addition, to verify the accuracy of the AAPH offline HPLC method, the TEAC assay was used to evaluate the antioxidant activity of phenolic compounds, and the results are shown in Table 2.

As shown in Table 1, at 65 °C, the percentage of peak area reduction of polyphenol compounds due to heating was between 3.03 and 23.92%; at 75 °C, the percentage of peak area reduction of polyphenols was between 4.32 and 34.80%; and when the temperature was increased to 85 °C, the percentage decrease in the peak area of polyphenols was between 4.65 and 39.20%. At 65 °C, we detected 16 compounds whose peak area reduction was due to the reaction with AAPH radicals; the percentage of peak area reduction was between 1.84 and 100%, and most of the peak area reduction rates were around 10%; at 75 °C, we detected a reduction in the peak area of 19 compounds. Due to the reaction with AAPH radical, the percentage of peak area reduction was between 1.46 and 100%, and most of the peak area decrease rate was approximately 50%; at 85 °C, we detected peak area reduction for 17 compounds, due to the reaction with AAPH radical, the percentage of peak area reduction was between 0.3 and 100%. The degradation rate of polyphenols and scavenging rate of AAPH radicals increased with increasing temperature. Affected by the decomposition products, 3,4-Dihydroxyhydrocinnamic acid and 4,4′-methylenediphenol, which have AAPH free radical scavenging activity, could not be analyzed when heated at 85 °C. Therefore, to ensure a lower polyphenol degradation rate and a more accurate analysis of compounds with AAPH radical scavenging activity, 75 °C is a suitable temperature.

### 3.2. Screening Antioxidations from Mixed Polyphenols Using AAPH, DPPH and ABTS Offline HPLC

As shown in Table 2 and Figure 2f,g,h, based on ABTS, DPPH, and AAPH free radicals, we used offline HPLC to screen antioxidants from six types and twenty polyphenol standards. Nineteen active compounds were screened using the AAPH offline HPLC method, and the peak area reduction rate was between 1.46 and 100%; 17 active compounds were screened by the DPPH offline HPLC method, and the peak area reduction rate was between 1.07 and 100%; and 18 active compounds were screened by the ABTS offline HPLC method, and the peak area reduction rate was between 6.74 and 27.92%. The results obtained by the AAPH offline HPLC method were consistent with those of TEAC and were more sensitive than the existing ABTS and DPPH offline HPLC methods. This is due to the particularity of AAPH radicals, which have shorter carbon chains, active double bonds, and are easier to react with polyphenols; moreover, this may be due to the relatively high temperature of the AAPH reaction system, which accelerates the production of AAPH radicals as well as increases the activity of phenolic compounds to attack radicals [19,20,21].

The antioxidant properties of phenolic acids are greatly influenced by molecular structural features, such as the presence of double bonds and the number and positioning of hydroxyl groups relative to the carboxyl functional group and carbon chain length [22,23]. The radical addition reaction to the double bond has a very important effect on antioxidant capacity [24]. Among the five polyphenols in the group of hydroxybenzoic acid and its derivatives, gallic acid, 2,5-dihydroxybenzoic acid, 4-hydroxyphenylacetic acid, and syringic acid were screened by AAPH offline HPLC, gallic acid, 2,5-dihydroxybenzoic acid, 3,4-dihydroxybenzoic acid, and syringic acid were screened by DPPH offline HPLC, and all the standards were screened by ABTS offline HPLC with antioxidant activity. In the AAPH offline HPLC method, 3,4-dihydroxybenzoic acid did not show antioxidant activity, but 2,5-dihydroxybenzoic acid with the same number of hydroxyl groups showed strong antioxidant activity because hydrogen bonds were formed between hydroxyl groups in 3,4-dihydroxybenzoic acid, and the antioxidant activity decreased. 4-Hydroxyphenylacetic acid has a Trolox equivalent of 0.74 ± 0.01 µM and cannot be screened out by the DPPH offline HPLC method, probably because only one hydroxyl group exists in its structure. Some studies have shown that 4-hydroxyphenylacetic acid has only a weak DPPH radical scavenging activity. In the hydroxycinnamic acid group and its derivatives, the Trolox equivalent of the six polyphenol standards was between 0.57 and 4.98% [25]. All standards were demonstrated to have antioxidant activity using AAPH and ABTS offline HPLC methods. However, 3,4-dihydroxyhydrocinnamic acid showed no antioxidant activity in the DPPH offline HPLC method. The number of hydroxyl groups and the number of double bonds in 3,4-dihydroxyhydrocinnamic acid and caffeic acid were the same, and the antioxidant activities obtained in Trolox equivalent, AAPH, and ABTS offline HPLC results were similar, but there was no antioxidant activity in DPPH offline HPLC, which may be related to free radicals related to the type. By reviewing the literature, we found no studies that have screened 3,4-dihydroxyhydrocinnamic acid for antioxidant activity using DPPH offline HPLC. Compared with p-coumaric acid, 2,4-dihydroxycinnamic acid showed stronger antioxidant activity in the results of Trolox equivalent and AAPH offline HPLC, which is due to one more hydroxyl group in 2,4-dihydroxycinnamic acid. The Trolox equivalent weight of the four polyphenol standards in the flavonoid group ranged from 0.71–8.16%. All standards were demonstrated to have antioxidant activity using AAPH and DPPH offline HPLC methods. However, (+)-catechin showed no antioxidant activity in the ABTS offline HPLC method. After a literature search, it was found that the ABTS free radical scavenging activity of (+)-catechin is low; hence, it is not easy to be screened out by the ABTS offline HPLC method [26,27]. The number of hydroxyl groups in ginseng was the lowest; therefore, apigenin had the weakest antioxidant activity in the results of Trolox equivalent, AAPH, and DPPH offline HPLC. In stilbenes, lignans, and other groups, all standards were demonstrated to have antioxidant activity using the AAPH offline HPLC method. 4,4′-Methylenediphenol had no antioxidant activity in the DPPH offline HPLC method, and 3,4-dihydroxy-L-phenylalanine had no antioxidant activity in the ABTS offline HPLC method. A literature search found that 4,4′-methylenediphenol has no DPPH free radical scavenging activity, and 3,4-dihydroxy-L-phenylalanine has no ABTS free radical scavenging activity. In conclusion, compared with the existing offline HPLC methods of ABTS and DPPH, the offline HPLC method based on the AAPH radical is more sensitive.

### 3.3. Screening Antioxidations from L. meyenii Extract Using AAPH, DPPH, and ABTS Offline HPLC

**Figure 3:**
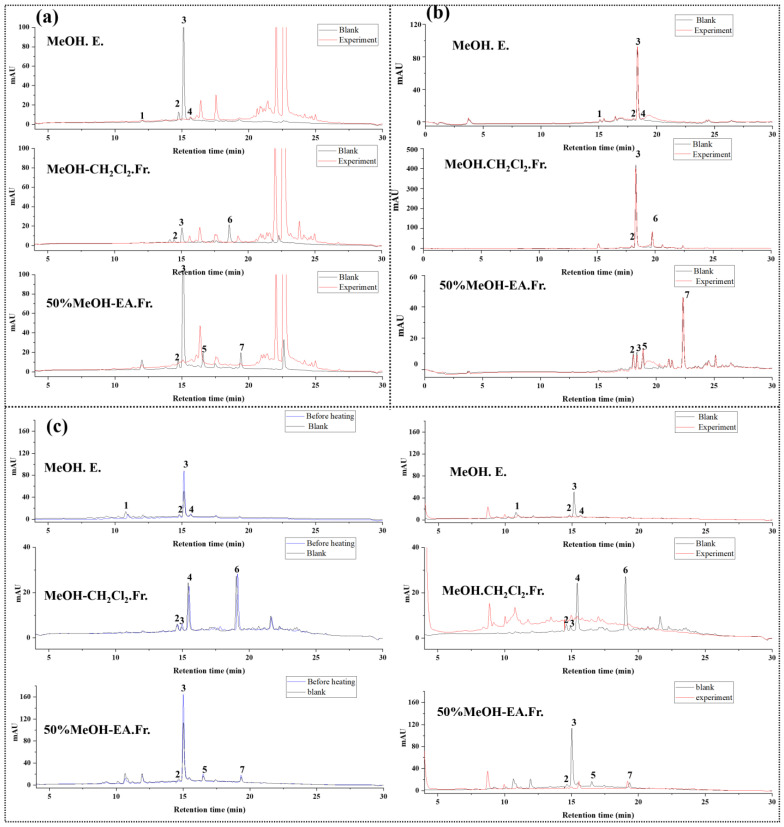
Results of screening antioxidants based on DPPH radicals (**a**), ABTS radicals (**b**), and AAPH radicals (**c**) from *L. meyenii* extracts and fractions.

In this study, we selected methanol extract (MeOH. E.), methanol extract chloroform fraction (MeOH-CH_2_Cl_2_. Fr.), and 50% methanol extract ethyl acetate fraction (50% MeOH-EA.Fr.), which contained all the compounds in *L. meyenii* extract, to investigate the antioxidations. In our previous studies, the components in *L. meyenii* extracts have been isolated and identified [14]. Therefore, in this study, we determined the components in *L. meyenii* extracts by retention time. As shown in Figure 3 and Table 3, we applied the AAPH offline HPLC method to *L. meyenii* extracts, compared the DPPH and ABTS offline HPLC methods, and used the TEAC method to calculate the Trolox equivalent to verify the accuracy of the AAPH offline HPLC method.

Through the TEAC assay, we used the standard curve method to calculate the Trolox equivalents of the seven main compounds in *L. meyenii* between 1.37 and 2.17 µmol, which have good antioxidant activity. In this study, the methanol extract of *L. meyenii*, chloroform fraction of the methanol extract, 50% methanol extract, and ethyl acetate fraction of the 50% methanol extract, which contains seven main compounds, were used as samples for antioxidant screening. In the AAPH offline HPLC method, the peak area reduction percentage of these seven compounds was between 17.56 and 100% because of the scavenging of AAPH radicals, and in the DPPH offline HPLC method, the peak area reduction percentage was between 12.26 and 100%. In the ABTS offline HPLC method, the peak area reduction percentages of these seven compounds ranged from 1.59 to 20.76%. Therefore, we believe that the results obtained by the AAPH offline HPLC method were consistent with those of the DPPH offline HPLC method and were more sensitive than those of the ABTS offline HPLC method. This is consistent with the Trolox equivalent calculated by the TEAC assay, which shows that the AAPH offline HPLC method developed based on AAPH free radicals is reliable and accurate and can be used for the screening of antioxidants in natural extracts.

## 4. Conclusions

In this study, we developed a novel offline HPLC method based on AAPH radicals for the antioxidant screening of 20 polyphenolic compounds and used the Trolox equivalent antioxidant capacity assay to evaluate their antioxidant activity. Compared with the existing offline HPLC methods for ABTS and DPPH, the offline HPLC method based on the AAPH radical is more sensitive. In addition, we applied this method to the *L. meyenii* extract and screened seven antioxidants, caffeic acid, hesperidin, rosmarinic acid, diosmin, methyl rosmarinate, diosmetin, and n-butyl rosmarinate, which are known antioxidants. Therefore, this study provides new insights into the screening of antioxidants in natural extracts.

## Figures and Tables

**Table 1:** Peak area reduction of polyphenols at different temperatures and results of screening antioxidants based on AAPH radicals.

Classification	Standards (Retention Time, min)	65 °C		75 °C		85 °C	
		Peak Area Reduction by Heating (%)	Peak Area Reduction by Radicals (%)	Peak Area Reduction by Heating (%)	Peak Area Reduction by Radicals (%)	Peak Area Reduction by Heating (%)	Peak Area Reduction by Radicals (%)
Hydroxybenzoic acids and derivatives	Gallic acid (8.11)	11.96	91.87	11.85	47.70	11.75	32.65
	3,4-Dihydroxybenzoic acid (14.19)	7.19	- ^1^	-	-	-	-
	2,5-Dihydroxybenzoic acid (19.48)	3.03	-	6.31	63.42	9.68	57.80
	4-Hydroxyphenylacetic acid (21.57)	-	11.03	-	23.32	-	0.30
	Syringic acid (25.16)	8.53	-	6.20	16.50	3.88	23.59
Hydroxycinnamic acids and derivatives	3,4-Dihydroxyhydrocinnamic acid (21.29)	19.38	13.46	-	59.11	4.65	-
	Caffeic acid (24.79)	12.22	6.10	10.24	53.15	8.25	69.63
	2,4-Dihydroxycinnamic acid (26.63)	10.47	8.84	17.18	31.20	15.75	38.07
	p-Coumaric acid (31.24)	10.43	1.84	8.88	1.46	7.34	8.02
	Sinapic acid (33.89)	11.94	44.30	4.32	10.63	16.91	38.16
	2-Hydroxycinnamic acid (38.28)	7.46	8.95	-	19.70	4.49	14.64
flavonoids	(+)-Catechin (20.57)	3.85	58.74	-	60.99	-	86.50
	Taxifolin (32.64)	10.07	15.47	29.18	19.65	-	82.72
	Quercetin (47.41)	23.92	98.21	31.56	98.48	39.20	99.01
	Apigenin (50.43)	11.33	6.73	9.88	14.33	9.80	23.46
Stilbenes	Resveratrol (39.93)	11.59	10.91	9.19	26.98	6.86	35.24
Lignans	Rosmarinic acid (40.68)	7.93	28.43	2.99	65.56	5.83	86.14
others	3,4-Dihydroxy-L- phenylalanine (4.60)	34.02	100.00	34.80	100.00	34.54	100.00
	4,4′-Methylenediphenol (46.23)	-	-	-	7.62	-	-
	Trolox (49.88)	-	69.97	-	4.97	-	27.92

^1^ “-” indicated that there is no peak reduction detected in the experiment.

**Table 2:** Peak area reduction of polyphenols at different temperatures and results of screening antioxidants based on AAPH radicals.

Classification	Standards (Retention Time, min)	Trolox Equivalents (TEAC, µmol)	AAPH	DPPH	ABTS
			Peak Area Reduction by AAPH Radicals (%)	Peak Area Reduction by DPPH Radicals (%)	Peak Area Reduction by ABTS Radicals (%)
Hydroxybenzoic acids and derivatives	Gallic acid (8.11)	6.37 ± 1.33	47.70	35.84	8.39
	3,4-Dihydroxybenzoic acid (14.19)	1.08 ± 0.62	-	3.88	7.78
	2,5-Dihydroxybenzoic acid (19.48)	2.33 ± 0.55	63.42	67.88	8.47
	4-Hydroxyphenylacetic acid (21.57)	0.74± 0.01	23.32	-	7.86
	Syringic acid (25.16)	2.57 ± 1.26	16.50	3.30	7.23
Hydroxycinnamic acids and derivatives	3,4-Dihydroxyhydrocinnamic acid (21.29)	2.54 ± 0.60 ^1^	59.11	- ^2^	8.54
	Caffeic acid (24.79)	1.99 ± 0.26	53.15	100.00	6.87
	2,4-Dihydroxycinnamic acid (26.63)	4.98 ± 1.03	31.20	1.07	7.32
	p-Coumaric acid (31.24)	1.15 ± 0.56	1.46	1.69	6.78
	Sinapic acid (33.89)	0.57 ± 0.27	10.63	34.30	8.68
	2-Hydroxycinnamic acid (38.28)	2.59 ± 1.84	19.70	4.69	7.11
flavonoids	(+)-Catechin (20.57)	5.47 ± 1.46	60.99	11.12	-
	Taxifolin (32.64)	2.98 ± 2.16	19.65	7.41	7.13
	Quercetin (47.41)	8.16 ± 0.91	98.48	45.40	12.37
	Apigenin (50.43)	0.71 ± 0.56	14.33	7.25	8.70
Stilbenes	Resveratrol (39.93)	2.96 ± 1.07	26.98	10.33	7.94
Lignans	Rosmarinic acid (40.68)	2.54 ± 1.24	65.56	4.81	6.74
others	3,4-Dihydroxy-L-phenylalanine (4.60)	1.15 ± 0.26	100.00	83.84	-
	4,4′-Methylenediphenol (46.23)	6.08 ± 0.87	7.62	-	14.87
	Trolox (49.88)	-	69.97	4.97	27.92

^1^ Trolox equivalents were shown as means ± SD, n = 3; ^2^ “-” indicated that there is no peak reduction detected in the experiment.

**Table 3:** Comparison of three offline HPLC methods of DPPH, ABTS, and AAPH of 7 compounds isolated from *L. meyenii*.

Classification	Standards	Trolox Equivalents (TEAC, µmol)	AAPH	DPPH	ABTS
			Peak Area Reduction (%)	Peak Area Reduction (%)	Peak Area Reduction (%)
Hydroxycinnamic acids	Caffeic acid (1)	1.37 ± 0.06 ^1^	100	62.79	13.71
Flavonoids	Hesperidin (2)	1.47 ± 0.25	72.23	61.67	13.77
	Diosmin (4)	1.87 ± 0.41	78.44	12.26	20.76
	Diosmetin (6)	2.17 ± 0.29	42.59	100	- ^2^
Lignans	Rosmarinic acid (3)	1.58 ± 0.04	100	97.48	1.59
	Methyl rosmarinate (5)	1.81 ± 0.37	17.56	100	4.68
	n-Butyl rosmarinate (7)	2.14 ± 0.42	23.38	81.49	100

^1^ Trolox equivalents were shown as means ± SD, n = 3; ^2^ “-” indicated that there is no peak reduction detected in the experiment.

## Data Availability

The data is included in the article.
